# Polyploidy: its consequences and enabling role in plant diversification and evolution

**DOI:** 10.1093/aob/mcac132

**Published:** 2022-10-25

**Authors:** J S (Pat) Heslop-Harrison, Trude Schwarzacher, Qing Liu

**Affiliations:** Key Laboratory of Plant Resources Conservation and Sustainable Utilization/Guangdong Provincial Key Laboratory of Applied Botany, South China Botanical Garden, Chinese Academy of Sciences, Guangzhou, 510650, China; University of Leicester, Institute for Environmental Futures, Department of Genetics and Genome Biology, Leicester, LE1 7RH, UK; South China National Botanical Garden, Chinese Academy of Sciences, Guangzhou, 510650, China; Key Laboratory of Plant Resources Conservation and Sustainable Utilization/Guangdong Provincial Key Laboratory of Applied Botany, South China Botanical Garden, Chinese Academy of Sciences, Guangzhou, 510650, China; University of Leicester, Institute for Environmental Futures, Department of Genetics and Genome Biology, Leicester, LE1 7RH, UK; South China National Botanical Garden, Chinese Academy of Sciences, Guangzhou, 510650, China; Key Laboratory of Plant Resources Conservation and Sustainable Utilization/Guangdong Provincial Key Laboratory of Applied Botany, South China Botanical Garden, Chinese Academy of Sciences, Guangzhou, 510650, China; South China National Botanical Garden, Chinese Academy of Sciences, Guangzhou, 510650, China; Center of Conservation Biology, Core Botanical Garden, Chinese Academy of Sciences, Guangzhou, China

**Keywords:** Polyploidy, whole genome duplications, structural variation, genome size, chromosome number, DNA and RNA sequencing, fluorescence *in situ* hybridization, flow cytometry, repetitive DNA, meiosis, ecology, speciation

## Abstract

**Background:**

Most, if not all, green plant (Virdiplantae) species including angiosperms and ferns are polyploids themselves or have ancient polyploid or whole genome duplication signatures in their genomes. Polyploids are not only restricted to our major crop species such as wheat, maize, potato and the brassicas, but also occur frequently in wild species and natural habitats. Polyploidy has thus been viewed as a major driver in evolution, and its influence on genome and chromosome evolution has been at the centre of many investigations. Mechanistic models of the newly structured genomes are being developed that incorporate aspects of sequence evolution or turnover (low-copy genes and regulatory sequences, as well as repetitive DNAs), modification of gene functions, the re-establishment of control of genes with multiple copies, and often meiotic chromosome pairing, recombination and restoration of fertility.

**Scope:**

World-wide interest in how green plants have evolved under different conditions – whether in small, isolated populations, or globally – suggests that gaining further insight into the contribution of polyploidy to plant speciation and adaptation to environmental changes is greatly needed. Forward-looking research and modelling, based on cytogenetics, expression studies, and genomics or genome sequencing analyses, discussed in this Special Issue of the *Annals of Botany*, consider how new polyploids behave and the pathways available for genome evolution. They address fundamental questions about the advantages and disadvantages of polyploidy, the consequences for evolution and speciation, and applied questions regarding the spread of polyploids in the environment and challenges in breeding and exploitation of wild relatives through introgression or resynthesis of polyploids.

**Conclusion:**

Chromosome number, genome size, repetitive DNA sequences, genes and regulatory sequences and their expression evolve following polyploidy – generating diversity and possible novel traits and enabling species diversification. There is the potential for ever more polyploids in natural, managed and disturbed environments under changing climates and new stresses.

## INTRODUCTION

Polyploidy, or whole genome duplication (WGD), is a ubiquitous feature of plant species evolution, and all groups of green plants (Viridiplantae, hereafter referred to as plants) have one or more events of WGD in their ancestry (see Soltis *et al.*, 2015; Alix *et al.*, 2017; van der Peer *et al.* 2017; Landis *et al.*, 2018; Chanderbali *et al.*, 2022). Indeed, the Amborella Genome Project (2013) showed that a WGD event preceded the origin of all angiosperms. About half of all plants – both crops and species in their native habitats – are recent polyploids with chromosome sets from two or more ancestors, and the ancestral diploid relatives are usually evident within the same or related genus. Polyploidy is arguably the most important force in plant speciation and genome evolution. Plants differ from the eukaryotic animal (David 2022) and fungal lineages, as well as viruses and bacteria, where WGD, although occurring sporadically through evolution, is typically a minor rather than widespread driving force. This Special Issue of *Annals of Botany* brings together research on polyploidy from fundamental to applied, with an emphasis on ecology and speciation and consequent advantages and disadvantages.

The advent of whole genome sequencing approaches, including survey sequence analysis of RNA or DNA, and whole genome assembly, has shown the signature of multiple WGD events throughout the evolutionary history of plants (reviewed by, for example, Wong *et al.*, 2020; Nieto Feliner *et al.*, 2022). These approaches complement cytogenetic and experimental hybridization methods, and show clearly gene duplications and shared regions of genomes. Combined with ecology and taxonomy, microscopy, genomics and modelling have provided valuable knowledge in understanding the occurrence and nature of polyploidy and its contribution to plant evolution. Knowledge of the mechanisms during the events leading to polyploidy, and their consequences, can be applied to evaluate the basis and measure the nature of plant biodiversity, and used for the development of new and improved crops to meet the challenges of the future.

## KEY TERMINOLOGY AND CONCEPTS IN POLYPLOID RESEARCH

### Polyploidy and the phrase ‘whole genome duplication’

**Fig. 1.**
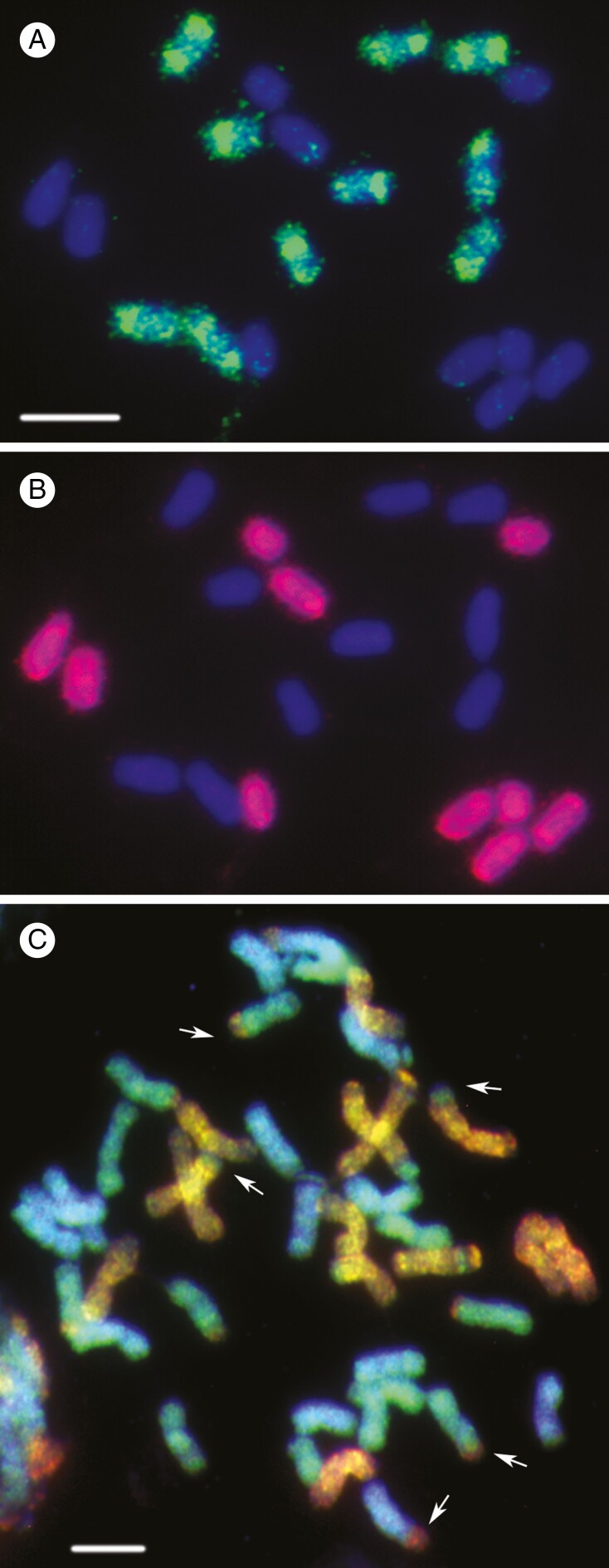
Fluorescence *in situ* hybridization (FISH) to identify genomes in polyploids. (A, B) Genomic *in situ* hybridization of a root tip metaphase of the allo-tetraploid water-starwart, *Calitriche platycarpa* (Plantaginaceae; 2*n* = 4*x* = 20), showing ten chromosomes hybridized with total genomic DNA from *C. cophocarpa* DNA probe (A; in green) and the remaining ten chromosomes hybridized with *C. stagnalis* DNA (B; in red); chromosomes were stained with DAPI (blue) showing the unlabelled chromosomes in each image. See Schwarzacher *et al.* (2016). Scale bar = 10 μm. (C) Metaphase chromosomes of *Avena sativa* (hexploid oat, 2*n* = 6*x* = 42) via FISH using mutiple genome-specific repetitive DNA probes, identifying the three genomes (A, B, C) in different colours and showing terminal translocations (arrows) between homeologous chromosomes (see Liu *et al.*, 2019, 2022). Scale bar = 5 μm.

Polyploidy and WGD have become widely used descriptions for multiplication of the chromosome numbers in a plant lineage, with little distinction between the two phrases. ‘Polyploidy’ often refers to evolutionarily more recent events, whether in the last few million years or up to a single generation. Its occurrence is usually clear in a taxon with a base chromosome number, *x*, and polyploids have multiples of this basic number. In autopolyploids involving a single species and chromosome number, or in allopolyploids originating from more than one taxon, the new chromosome number is based around the sum of chromosome numbers in the multiple ancestral species (Fig. 1A, B) although, in many polyploids, chromosomes may be lost or gained. The ancestors of allopolyploids are most often related species that have themselves diverged from a common ancestor, and the diverged chromosomes with the same set of genes are referred to as homoeologues, rather than the homologous pairs from the parents of a diploid or autopolyploid. ‘Whole genome duplication’ has been used frequently for events occurring many million years ago (including where the ancestral genomes involved are not extant or chromosome numbers have changed extensively), and in studies making comparisons of sequence assemblies. These are referred to as ancient or palaeo-polyploids (or having ancient WGD events), while recent polyploids would have WGDs since the genus or closely related genera diverged (see e.g. Mayrose *et al.*, 2011). Nevertheless, WGD may be used to refer to new lineages and experimental hybrid products (Mattingly and Hovick, 2022). In this review, the term ‘polyploidy’ is used regardless of the original author’s preference. Polyploidy occurring in specific cells or tissues of an individual, sometimes part of development or differentiation in a single plant (as in the endosperm, suspensors of the egg apparatus, or sometimes leaf or tapetum cells of angiosperms), is not considered here.

### Genome

There is no concise definition of ‘genome’, and, indeed, the term is used in multiple ways in different publications, so the context must be known. Whole nuclear genome sequencing and assembly will deliver the DNA sequences (including genes, regulatory, structural and repetitive sequences) of a haploid chromosome set from the nucleus of a plant (as found in a gamete) – the genome. However, cells in plants of a hexaploid species, such as bread wheat (*Triticum aestivum*) or cultivated oat (*Avena sativa*, see Fig. 1C; Liu *et al.*, 2019), can be described as having one genome in each nucleus, including all the DNA molecules; or two genomes, one each from the mother and father; or three genomes, one from each of the three ancestral species (sometimes referred to as sub-genomes); or six genomes, each representing one haploid set of chromosomes from the ancestors; or, indeed, these numbers plus two for the mitochondrial and chloroplast genomes. A cell might be regarded as having hundreds of genomes if all those of individual chloroplasts, mitochondria and even commensal or pathogenic viruses and bacteria are included. Other studies of genomes will include DNA modifications, in particular cytosine methylation, and chromatin packaging with various histones.

### Chromosome number

In an angiosperm, the cells of the sporophyte, the plant that will give rise to the spores, are described as having a chromosome number of 2*n*, regardless of whether the plant is diploid, triploid, tetraploid or higher ploidy. After meiosis the haploid (1*n*) products (‘spores’) develop into gametophytes (pollen or embryo sac in angiosperms, with only a few mitotic divisions) and produce the 1*n* gametes (in angiosperms, the generative pollen nucleus and egg cell) that fuse upon fertilization to the diploid (2*n*) zygote that develops into the sporophyte (embryo). The gamete chromosome number is expressed as *n*, while the basic chromosome number is expressed as *x*, denoting the ploidy level, such as 2*x*, 3*x*, 4*x* or higher. Thus, for example, bread wheat or oat (Fig. 1C) would be described as 2*n* = 6*x* = 42. Nevertheless, the basic chromosome number of a group including various polyploids can be difficult to infer (see Tomaszewska *et al.*, 2022 with *Urochloa* diploid and polyploid species; and Elliot *et al.*, 2022 in *Schoenus*) with substantial chromosome fusion, fission and other rearrangements, where chromosome-level genome assemblies have proved powerful in identifying events, for example in the evolution of chromosomes in *Petunia* diploid and hybrid species (Bombarely *et al.*, 2016) and the whole genome assemblies of *Buxus* (Buxales) and *Tetracentron* (Trochodendrales) showing independent WGDs and the ancestral genome structure of early eudicot diversification (Chanderbali *et al.*, 2022).

## POLYPLOIDY, SPECIATION AND EVOLUTION

**Fig. 2.**
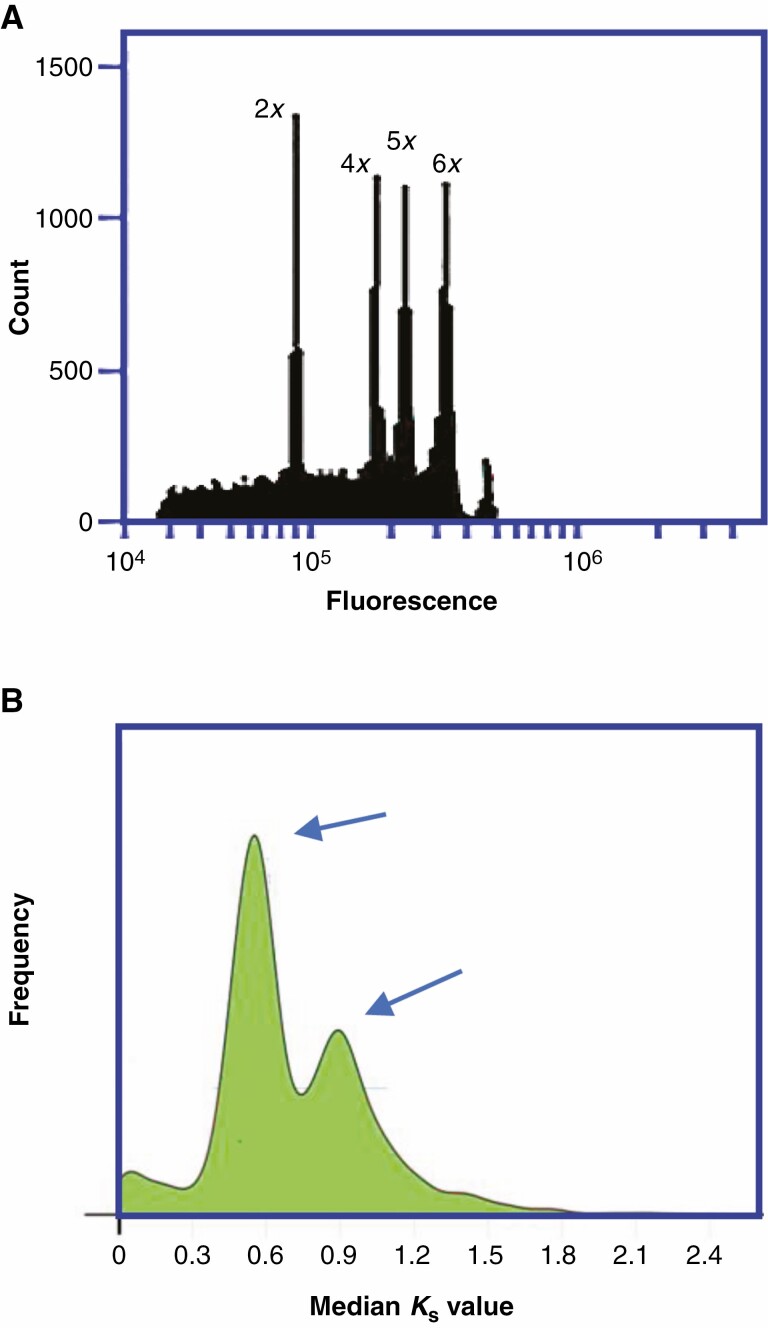
Determining ploidy. (A) Flow cytometry measurements of propidium iodide-stained nuclei. Overlay of graphs from diploid (2*x*), tetraploid (4*x*), pentaploid (5*x*) and hexaploid (6*x*) accessions of *Urochloa bryzantha* group species (see Tomaszewska *et al.*, 2022). (B) Density distributions of synonymous substitutions (*K*_s_) between genes in paired collinearity gene groups (orthologues) within the genome of *Ensete glaucum*. Peaks indicate whole-genome duplications (WGDs, arrows). (Figure adapted from Z. Wang *et al.*, 2022).

Many of the papers in this Special Issue provide examples of ongoing evolution in chromosome number and genome size in polyploids (e.g. *Schoenus*, Elliot *et al.*, 2022; *Urochloa*, Tomaszewska *et al.*, 2022; *Nicotiana*, Chase *et al.*, 2022; *Allium*, G. Wang *et al.*, 2022), often involving amplification or elimination of retroelement and repetitive DNA sequences as well as of genes. For chromosome analyses, cell spreading techniques and karyotyping are powerful tools in showing recent polyploidy in species by counting chromosomes and establishing the basic chromosome number (*x*) in a group, and the polyploids with near-multiples of it. Chromosome analysis has been used successfully since the 1930s (Darlington, 1939) and as shown by the examples in this issue and in Fig. 1 is essential today. The measurement of genome size, via flow cytometry in correlation with ploidy where there is no or limited loss or gain of DNA, is another important cytogenetic method to measure ploidy, and is often easier than making chromosome preparations, showing distinct peaks with different numbers of genomes (Elliott *et al.*, 2022; Pomies *et al.*, 2022; Semberova *et al.*, 2022; Tomaszewska *et al.*, 2022; Fig. 2A). However, care needs to be taken as chromosome numbers and genome size may change (independently or dependently) post-polyploidization, through sequence loss and gain, chromosome elimination, or chromosome fusions and fissions. Additional to cytogenetic examinations, the program ChromEvol (Glick and Mayrose, 2014), if a draft phylogeny and chromosome number data for the species involved are available, has proved valuable to infer locations and types of chromosome number changes along the phylogeny of plants (Chase *et al.*, 2022; Elliott *et al.*, 2022).

Examination of meiotic chromosome pairing is helpful in polyploids, but is influenced by genes that ensure exclusive pairing of homologues. To avoid formation of multivalents at meiosis, with subsequent chromosome non-disjunction and infertile gametes, most polyploids have developed genetic mechanisms, such as the Ph-locus in wheat, to ensure regular chromosome pairing at meiosis occurs (Singh *et al.*, 2019), and diploid behaviour is restored in the polyploid. This then leads to diploidization of polyploids that makes them successful new species (Li *et al.*, 2021), although Levin (2021) has speculated that limited numbers of propagules from new polyploids may limit establishment of new polyploid populations. Alternative mechanisms of propagation, involving either apomictic seed production or vegetative propagation, enable reproduction of taxa without regular meiosis, and allow taxa with odd numbers of ancestral genomes: for example, triploid dessert banana, *Musa acuminata* (Heslop-Harrison and Schwarzacher, 2007); heptaploid dog rose, *Rosa canina* (Hertzklotz and Ritz, 2017); and triploid saffron, *Crocus sativus* (Schmidt *et al.*, 2019). Important examples of vegetatively or seed-propagated triploids discussed in this Special Issue are aspen trees (*Populus tremuloidies*; Eisenring *et al.*, 2022) and palms (Pomies *et al.*, 2022), but also some sexual or mixed apomictic–sexual systems, such as *Urochloa* forage grasses, including multiple ploidies (Tomaszewska *et al.*, 2022). However, beyond chromosomal explanations for sterility of polyploid hybrids, morphology (Šemberova *et al.*, 2022), ecological niche differentiation (Chase *et al.*, 2022) or isolation of populations on islands (Joshi *et al.*, 2022) lead to separation of polyploid lineages allowing evolution independent from the original diploid species, diversification and speciation.

Backcrossing to a suspected diploid can clearly show the presence of multiple genomes with meiotic pairing in the F_1_ hybrid between chromosomes of one of the genomes forming bivalents while remaining chromosomes are seen as univalents. Examples include diploid and tetraploid relatives of wheat in the genus *Dasypyrum* (Galasso *et al.*, 1997; see also N. Wu *et al.*, 2022). The existence of polyploidy can also be inferred by the presence of aneuploids with missing chromosomes: diploid species with missing chromosomes are almost never viable (notwithstanding the fact that haploid plants occur regularly). Early suggestions that *Zea mays* was a tetraploid (palaeo-tetraploid) actually came from the discovery of lines with 2*n* = 4*x* − 1 = 19 chromosomes derived from the euploid 2*n* = 4*x* = 20.

*Erysimum* (Brassicaceae) provides an excellent example of a genus where evolution in a small area, the southern Iberian Peninsula, can be followed from a dynamic glacial refugium where range expansions and contractions have led to cycles of sympatry and isolation. Osuna-Mascaro *et al.* (2022) show that extensive polyploidization, interspecific hybridization and introgression have driven diversification, with evidence for frequent and on-going hybridization and introgression across ploidy levels, including indications of ancestral and extinct taxa. The whole-genome effects of hybridization and interplay between hybridization and introgression show the importance of polyploid events in complex scenarios of plant evolution.

Similarly, isolated populations, often endemic to islands, are of particular interest because they can show evolutionary events consequent to the small population sizes, with different selective pressures from those in larger ecosystems. Joshi *et al.* (2022) investigate genome dynamics in a polyploid Malvaceae species (*Plagianthus regius*), finding that the polyploid island species shows chromosomal stasis, although there is evidence for small-scale processes including loss of ribosomal repeats and copies of the *GBSS1* (granule bound starch synthase) gene.

### Genomic signatures of polyploidy

**Fig. 3.**
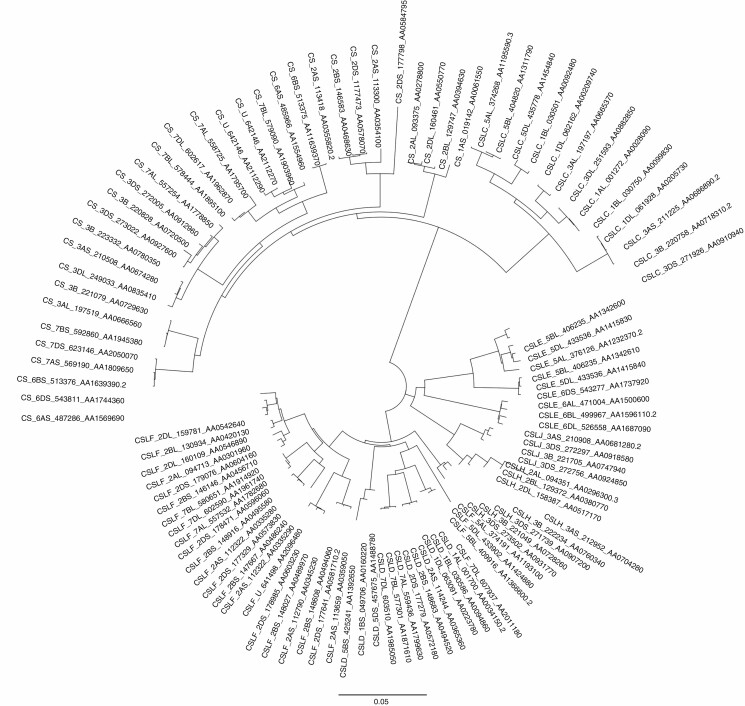
Multiple copies of genes are detected in polyploids. A neighbour-joining tree (bar represents 0.05 changes/site) (no outgroup, Jukes–Cantor distances) of cellulose synthase-like (Csl) genes in the genome of hexaploid wheat (modified after Liu *et al.*, 2022). Most genes are present in three copies, one from each of the A, B and D genomes (orthologous genes in the ancestral diploid species). In a few cases, genes have been deleted (or were not detected due to substantial sequence divergence) in one or two genomes, or additional copies have arisen by segmental duplications in either the diploid ancestors or the hexaploid.

Genome sequencing and isolation of orthologous genes reveals the presence of multiple copies of genes (see Fig. 3); variation in sequence, including duplications and allelic variation between the parental genomes, can occur in the ancestral species, and new functions can be gained (see below). However, gene or chromosome segment duplication can give more copies of genes, unrelated to polyploidy, and there may be chromosomal deletions, recombination or mutation that make WGD harder to detect in either the polyploid or the ancestors. For example, analysis of duplicated blocks of genes has shown that rice contains evidence of ancient aneuploidy having undergone duplication of a whole or large part of one chromosome rather than going through a polyploid event early in its evolution as previously thought (Vandepoele *et al.*, 2003). Overall, however, genome-wide sequencing methods including RNA-seq (RNA, normally poly-A mRNA, sequencing) combined with analysis of SNPs (single nucleotide polymorphisms) have proved powerful and were used to show both the presence of multiple alleles and their origin in comparison with the ancestral diploids in tetraploid coffee, *Coffea arabica* (Combes *et al.*, 2022). In an alternative approach, high and low-depth survey sequencing can calculate synonymous divergence (*K*_s_) frequencies and thus detect ancient WGDs (Fig. 2B), often showing several rounds of palaeopolyploidy events, for example in kiwi fruit (*Actinidia*) and its relatives (Shi *et al.*, 2010) and the ornamental *Petunia* species (Bombarely *et al.*, 2016). Here, Zhao *et al.* (2022) show multiple WGDs events in the *Arum* family, Araceae, finding that net diversification rates are enhanced after the WGD event, which is helpful in discovering the phylogeny of the group.

High-quality, chromosome-scale, assemblies of genomes allow analyses of homoeologous and orthologous gene identification that can be displayed at the chromosome level by connecting groups of genes, thus showing regions of conserved synteny. The application of these bioinformatics approaches using appropriate parameters for similarity, and displayed using Synvisio or within Circos plots in many genome assembly papers, clearly shows the presence of duplications or higher levels of similarities in the order of genes, and both small duplications and WGDs as well as genome expansions and structural variations are evident: examples include the WGDs found in Musaceae species (D’Hont *et al.* 2012; Z. Wang *et al.*, 2022), and in the more recent allopolyploid peanut, *Arachis hypogaea* (Bertioli *et al.*, 2013) and *Avena* diploids and polyploids (Liu *et al.*, 2022). WGDs can be inferred from fragmented (‘draft’) genome assemblies, particularly in a comparative context (e.g. in 1000 plant transcriptomes, see Wong *et al.*, 2020), although in restricted analyses, homoeologous copies of genes may be collapsed to a single sequence, or alternatively alleles of homologues or simple gene duplications may incorrectly suggest WGD [see above and pointed out by Z. Wang *et al.* (2022)]. G. Wang *et al.* (2022) discuss the large genomes in three *Allium* clades, showing evidence for gradual change, with various features of contraction, expansion and stasis, followed by adaptive radiation. The authors suggest that intense selective pressures in the Qinghai–Tibetan Plateau may be driving the evolutionary changes with a tendency for polyploids to grow at higher altitude.

Repetitive DNA represents the majority of the DNA in most species, and includes short sequence motifs that are repeated in tandem arrays at one or multiple loci, or dispersed repeats that are often related to transposable elements (see Zhang *et al.*, 2022). Repetitive DNA is usually the most variable and rapidly evolving components of the genome, often showing substantial differences in both sequence and copy number of individual motifs between species (Heslop-Harrison and Schwarzacher, 2007). Hence, repetitive sequence analysis is able to identify divergent ancestral genomes in polyploids, through identification in individual clones or PCR amplification products, and through genome-wide surveys to identify genome-specific motifs. If the ancestral genomes differ widely in repetitive sequence, *in situ* hybridization with total genomic DNA (GISH or genomic *in situ* hybridization) can successfully distinguish chromosome of different ancestral origins (see N. Wu *et al.*, 2022; Fig. 1A, B).

Characterization of the repetitive component of the genome is discussed by Zhang *et al.* (2022) and Tomaszewska *et al.* (2022) using unassembled survey sequence reads, in the case of *Urochloa* (*Brachiaria*) identifying repetitive DNA sequence motifs that were specific to, or excluded from, some of the accessions, showing the nature of evolution leading to differentiation of the ancestral genomes. Genome-specific repetitive sequences can be used as probes for chromosomal *in situ* hybridization (e.g. Bertioli *et al.*, 2013; Liu *et al.*, 2019; Fig. 1C) or to determine abundance in sequences reads, allowing reconstruction of the phylogeny of species in the genus and genome composition of polyploids. In *Urochloa*, the results can be applied in forage grass improvement and enable exploitation of wider pools of germplasm. Where high-quality genome assemblies are available, massive pools of low-copy sequences can be synthesized and used to identify homologous pairs and homeologous groups of chromosomes by *in situ* hybridization, as has been shown by Agarwal *et al.* (2018) and Šimoníková *et al.* (2019).

## ARE POLYPLOIDS SUCCESSFUL?

The advantages of polyploidy, through allowing access to multiple alleles and copies for each gene (Fig. 3), could be large, by essentially fixing heterozygosity and ‘hybrid vigour’, bringing together genes from different species, and increasing diversity (e.g. te Beest *et al.*, 2012; Landis *et al.*, 2018; Zhang *et al.*, 2022). In tetraploid coffee (*Coffea arabica*), Combes *et al.* (2022) combined biosynthetic pathways bring together advantageous genes (either ecologically or economically). The presence of genome modifications in the tetraploid including exchanges between homeologous chromosomes and, later, duplicate gene evolution involving gene conversion and homeologue silencing, could have played a role in its stabilization and subsequent diversification. A similar situation is found in the recent allopolyploid peanut, where Bertioli *et al.* (2013) show that after polyploidization, the genomes have evolved through sequence deletions, activation of mobile-elements and homeologous exchange between corresponding ancestral chromosomes, all contributing to the success of the crop.

In a polyploid, copies of a duplicated gene are released from selection pressures, so one gene may retain a function while others are free to mutate and gain new functions: mutations may not be lethal, and indeed Joshi *et al.* (2022) show how genes can be lost in polyploids compared to their diploid progenitors. As an alternative counter-argument, the additional burden of replicating and controlling the multiple genomes in polyploids, and need for different mechanisms to produce the next plant generation potentially without opportunity for recombination, might be so great that polyploids are rare. As pointed out by Levin (2021) establishment of polyploid populations then becomes difficult.

Interestingly, the world’s four major crop species (FAOstat, 2022), equally successful under intense selection, exploit different forms of genome expansion through duplication or polyploidy: wheat is a hexaploid albeit with diploid-like behaviour at meiosis, rice is a diploid but incorporates a large chromosome duplication; maize is an ancient tetraploid with two ancestral diploid genomes; and sugarcane cultivars are high polyploids derived from interspecific hybrids (2*n* = ~12*x* = ~120) with recombinant chromosomes (Pompidor *et al.* 2021). Among all crops, both diploid and polyploid species are common. Plants in natural environments, with different selective pressures from crops, similarly include both diploids and polyploids in angiosperms (*Allium*: G. Wang *et al.*, 2022; *Nicotiana*: Chase *et al.*, 2022) and ferns (Fujiwara *et al.*, 2022), as well as Lycophytes (Wood *et al.*, 2009). However, polyploidy is rare in gymnosperms (a group nested within the phylogenetic tree of plants) with only the genera *Ephedra* and *Juniperus* having abundant polyploids (Ickert-Bond *et al.*, 2020).

Fujiwara *et al.* (2022) present a comprehensive analysis of genome sizes and chromosome numbers across 233 fern species, more than doubling the number studied previously and including all taxonomic orders and half of the fern genera. Among the 10 000 fern species, one has the highest known chromosome number (2*n* = ~1440 in *Ophioglossum reticulatum*), and probably a very high frequency of polyploidy-enforced speciation events (Wood *et al.*, 2009). There were remarkable differences in genome size (1C), varying 630-fold from 234 Mb (*Salvinia cucullata*) to 147 291 Mb (*Tmesipteris obliqua*), with a 109-fold range in average DNA amount per chromosome. Study of the dynamics of genome evolution and diversification has shown that evolutionary trends in genome size, monoploid genome size (average size of the ancestral diploid genomes in a polyploid) and chromosome size correlated with the rate of evolution, mainly explained by the high frequency of polyploidy, high chromosome numbers and high proportion of repetitive DNA. Diversification of fern lineages has been shaped by the enhanced rate and dynamics of genome evolution, including but not restricted to polyploidy (Fujiwara *et al.*, 2022), and may have promoted the success of some recently diverging lineages. Complementing these results, a high-quality assembly of the tetraploid homosporous fern *Adiantum nelumboides* shows the species has a high ancestral chromosome number in the young polyploid, with a lack of substantial genome downsizing and dominance of genic diploidization compared to its diploid ancestors (Zhong *et al.*, 2022).

### Environmental adaptation of polyploids

Comparisons between diploid and polyploid natural taxa feature in many papers in this Special Issue, with notable differences in their ranges (even if polyploids tend to have more expanded ranges than their diploid ancestors; see Liu and Wang, 2022), and with ecological advantages; adaptation of polyploid species has been particularly studied with respect to drought and water stress. Chase *et al.* (2022) found lower chromosome numbers in *Nicotiana* species from drier areas of Australia, suggesting that this stress favours lower chromosome numbers with perhaps less recombination, and speciation is linked to radiation of species into novel habitats, with a background of diploidization. On the other hand, in the South African genus *Schoenus*, Elliot *et al.* (2022) found polyploid species occurred in drier regions and more variable climatic regions compared with the diploid species; and polyploid *Allium* were found preferentially at higher altitude with harsher environments (G. Wang *et al.*, 2022). Zhao *et al.* (2022) suggest that, within the Araceae (*Arum* family), polyploidy plays an important role in the evolution of adaptations to tropical, terrestrial environments in the True-Araceae clade through diversification and functional enrichment of genes, although in the Proto-Araceae group and the lemnoid clade, the consequences of WGD related to adaption to aquatic environments were less evident. From field studies, Eisenring *et al.* (2022) quote reports that triploid aspen (*Populus tremuloides*) are larger and grow faster than diploids and they show that triploid trees were more drought resilient than diploids (producing 35 % more new tissue during recovery). However, despite triploids having higher foliar defence, photosynthesis and rubisco activity, for other traits (including tree growth under non-stressed conditions), genotypic and population differences, unrelated to ploidy, explained the variation observed. Mattingly and Hovick (2022) take an ingenious experimental approach to test the long-standing hypothesis that polyploids have increased phenotypic plasticity compared to their diploid counterparts. Using diploid and autotetraploid material of *Arabidopsis thaliana*, they found most adaptive characteristics were similar between the 2*x* and 4*x* lines, and the result showed that polyploidy alone could in occasional cases increase phenotypic plasticity under stress, but the effects were genotype- and environment-dependent and were only one aspect of adaptation.

Another widely considered feature of polyploids is gigantism: in crops, selection for a large size of the harvested parts is an important domestication trait. Polyploidy has been correlated with gigantism, for example an increase in fruit size in kiwi, *Actinidia chinensis* (J. H. Wu *et al.*, 2012). Pomies *et al.* (2022) characterized oil palms (*Elaeis guineensis*) in a breeding programme and identified ‘giant palms’ based on 11 phenotypic traits: half of the giant palms were triploids, originating from 2*n* gametes, although field experience suggests the bunches will have poor fruit set so will not be useful for breeding. Notably too, half of the ‘giant palms’, rigorously defined by a principal components analysis of 21 characters, were diploids, suggesting alternative mechanisms are also responsible for the increase in size.

### Polyploidy, gene expression and genome dominance

Gene or trait expression in polyploids involves both gene allele variants and control of genes between the ancestral genomes that have come together (van der Peer *et al.*, 2017). Liu and Wang (2022) review some of the evidence that polyploidy generates novel genetic changes including rapid and non-random gene loss, and silencing and activation of DNA sequences, and they review critically claims of dominance of ancestral genomes in polyploids. However, they conclude that there is no strong evidence for ‘genome dominance’, with widespread differences in gene expression between ancestral genomes in polyploids. This is contrary to the view that there are conflicts among the genomes and that one generally retains more, highly expressed, genes (see Jones and Pasakinskiene, 2005). Polyploids, however, can be viewed as peacemakers (see Alix *et al.*, 2017) or as discussed by Liu and Wang (2022), any differences leading to genome plasticity probably reflect differences in the genetics of the progenitors.

High-throughput RNA-seq gives unprecedented detail about which allele of a gene is being expressed across the whole genome, and, using appropriate SNPs, allows correlation with the parent-of-origin. The ancestral genomes of polyploids often differ in gene number (e.g. Zhao *et al.*, 2022), which may affect gene expression and epigenetic alteration of the control of gene expression depending, or not, on the interactions of the ancestral diploid genomes. In tetraploid coffee (*Coffea arabica*), Combes *et al.* (2022) discuss the evidence from previous studies showing no preferential expression of one genome over the other in tetraploids, and consider that patterns of gene expression inheritance and expression level dominance are determined by regulatory divergences between parental alleles. Specifically concentrating on genes involved in caffeine biosynthesis (influencing caffeine content) by RNA-seq and SNP analysis during the gene expression during maturation of seeds and in leaves, their comparison of the tetraploid and the two progenitor species showed no preference in expression of one ancestral genome. Some homoeologue expression bias (HEB) observed in 30 % of the genes was due to differences in gene expression inherited from the diploid progenitor species, and they conclude that genes from both genomes are balanced with expression of both homoeologues, with interoperability of regulatory networks from the ancestral diploids.

## WHAT COMES AFTER POLYPLOIDY

After the polyploid formation event, polyploids and interploid hybrids may become isolated from their progenitors by pre- and post-zygotic reproductive barriers; sterility due to irregular meiotic pairing and multivalent formation; and the problems caused by controlling multiple genes in new genomic environments (see section ‘Polyploidy, speciation and evolution’). Semberova *et al.* (2022) show, in *Campanula rotundifolia*, how reproductive isolation is also caused by different cytotype morphological differences and, importantly, by morphological variation driven by ploidy differences and environmental adaptation, most notably with the environmental niche shift in the higher polyploids. However, the effects of ploidy and morphological differences appear to vary between species: in *Erysimum* (Brassicaceae), Osuna-Mascaro *et al.* (2022) show that hybridization occurs regardless of flower colour and ploidy level. Once polyploids are established and become isolated, divergence and speciation become possible, although a single species may be present at several different ploidy levels (discussed in many papers in this Special Issue).

In the plant breeding context, the diversity of different genomes in a species group can be exploited through interploidy crosses. N. Wu *et al.* (2022) show that chromosomal diversity in the diploid wheat-relative *Dasypyrum villosum* (2*n* = 2*x* = 14) can function as a resource for improvement of tetraploid durum wheat (2*n* = 4*x* = 28) through crossing and chromosome doubling, generating a hexaploid amphidiploid (2*n* = 6*x* = 42). The authors show how these interploid crosses enable chromosome engineering through backcrosses and recombination, introducing novel characters into wheat, where the hexaploid nature and reproductive isolation has restricted the diversity available in the wider germplasm pool available in the tribe Triticeae. Tomaszewska *et al.* (2022), working with the tropical forage grass *Urochloa* (*Brachiaria*), suggest how knowledge of the natural diversity in the species complex can be exploited through well-defined programmes of intercrossing, assisted by knowledge of the genomes of diploids and the ancestral genome composition of polyploid taxa. In summary, the ability to generate polyploids is an important route to exploit biodiversity and widen the gene pool available to breeders.

Post-polyploidization, chromosome number sometimes reduces during restoration of pseudo-diploid chromosome pairing (Chase *et al.*, 2022; Tomaszewska *et al.*, 2022). Chase *et al.* (2022) specifically address what happens to genome size and chromosome number after polyploid formation, using 46 allotetetraploid species of *Nicotiana* that radiated in the centre of Australia. Chromosome number, genome size and ploidy show a complex relationship and the authors aim to discriminate the effects of genome size change from chromosome number change. Notably, within one section, species with fewer than 20 pairs of chromosomes show stable or increasing genome size, while species with higher chromosome numbers show decreasing genome size. Their analyses detect distinctive evolutionary dynamics in various sections of the genus, which helps reconstruction of the speciation history of the group, independently of gene-level and SNP analyses.

## CONCLUSIONS

Polyploidy plays a major role in speciation and diversification of almost all plants, bringing together new gene combinations and leading to reproductive isolation. However, as we discuss above and the paper in this Special Issue show, there are rather few ‘rules’ and it remains hard to find general patterns of evolution dependent on, or consequential from, polyploidy. One major plant group, the gymnosperms, even has very few polyploids, and the occurrence of polyploidy seems to be of little consequence, so a major plant group, dominant in many ecosystems, is successful without polyploidy. However, the survey of ferns by Fujiwara *et al.* (2022) shows the widespread occurrence of polyploidy in this group, extending and complementing the work in angiosperms.

Generalizations regarding the impact of polyploidy have been made, including increased phenotypic plasticity, leading to stress tolerance, and gigantism. However, it is not clear that these apply universally, and it seems that polyploidy is only one force of many in plant evolution: polyploids are equally successful as diploids among both crops and in the natural environment. Many publications indicate that global extinction rates are currently very high, perhaps a consequence of climate change. Levin (2019) considered that species diversity is likely to decline as a result of climate change while speciation will generate new species, primarily through auto- and allopolyploidy. At the Cretaceous–Tertiary transition, a time of mass extinction of 75 % of extant species, many new polyploid species (WGDs) appeared (Soltis *et al.*, 2015). Is there going to be an explosion of polyploids in response to climate change? Or will increased numbers of polyploid species be a response to the essentially global management and selection of not only crops – and their commensals, weeds – but all other areas of the world by humans?
